# Unconscious associations between stressor type and ability to cope: An experimental approach using ancient and modern sources of stress

**DOI:** 10.1111/bjhp.12587

**Published:** 2022-02-21

**Authors:** Evangelos Katsampouris, Julie M. Turner‐Cobb, Rachel Arnold, Julie C. Barnett

**Affiliations:** ^1^ Department of Psychology University of Bath UK; ^2^ Department of Psychology Bournemouth University UK; ^3^ Department for Health University of Bath UK

**Keywords:** ancient, coping, experiment, implicit associations, modern, stress

## Abstract

**Objectives:**

Work has emerged that suggests it is salient and feasible to include a chronological approach to the taxonomy of stress. The ability to make an explicit distinction between ancient stressors (AS) and modern stressors (MS) has been reported in young and older adults; AS have been associated with greater ability to cope and MS with poorer health outcomes. Whether these explicit distinctions exist at an implicit, unconscious level, has yet to be determined.

**Design:**

A quantitative design employed a computer‐based Implicit Association Test (IAT) to examine implicit associations between AS/MS and coping appraisal.

**Methods:**

One hundred adults (75 females) aged 18–58 years (*M* = 28.27 years, *SD* = 10.02) completed the AS/MS IAT, to compare reaction time (RT) and accuracy between consistent pairs (AS/ability to cope; MS/inability to cope) and inconsistent pair responses (AS/inability to cope; MS/ability to cope); followed by an explicit self‐report questionnaire.

**Results:**

Repeated measures ANCOVAs, controlling for sex and age, revealed significant main effects of faster RT and higher accuracy in responses for consistent than inconsistent pairs. Adult participants made implicit associations indicating an unconscious AS and MS distinction. Using the *D* algorithm, a univariate ANCOVA and independent *t*‐tests found that males, compared to females, showed a stronger implicit preference for consistent than inconsistent pairs.

**Conclusions:**

Findings suggest an implicit association between ancient and modern stressors and perceived coping ability. Utilizing a chronological taxonomy for understanding evolutionary origins that drive individual’s responses to stress has implications for developing effective coping strategies to improve health outcomes.

**Statement of contribution**

***What is already known on this subject?***

- Psychosocial characteristics enable to profile ancient and modern stressors along a continuum.
- Adaptive psychophysiological coping processes enable adults to better withstand ancient than modern stressors.
- Modern, rather than ancient, stressors might have a greater impact on adults’ health.

***What does this study add?***

- A deeper understanding of the distinction between ancient and modern stressors.
- An experimental paradigm to assess adults’ implicit associations between stressor type and coping ability.
- A novel stress taxonomy is suggested based on an ancient/modern stressor chronology.

## Background

Stress is considered as a complex concept that has been explicitly assessed at both subjective and objective levels via psychosocial and physiological measurement. An important and often overlooked theoretical perspective in understanding stress is the social evolutionary categorization of stressors as ancient or modern. Ancient stressors (AS) have been considered those life events that people have had to cope with since the beginning of the time (e.g., the death of a close relative). Modern stressors (MS) are relatively evolutionarily newer (e.g., being unemployed) and require from individuals to spend more time and energy to cope. Evidence suggests that established adaptive psychophysiological coping mechanisms enable individuals to cope better with AS, given they have been an integral part of human evolution, compared to more MS that require greater adaptation and coping ability (Schreier & Evans, 2003).

Recent work has assessed the feasibility of explicitly distinguishing between AS and MS, using mixed methods, resulting in the development of a novel stress taxonomy that observes this ancient/modern chronology, enabling a distinction between AS and MS based on five psychosocial stressor characteristics of coping, experience, manageability/expectedness, duration, and type of the stressor (Katsampouris, Turner‐Cobb, Barnett, & Arnold, 2020). In young and older adults, the life events of death/bereavement, movement, health/illness of others, and social/interpersonal arguments were designated as AS and associated with greater ability to cope, whilst the life events of unemployment, financial problems, health/illness of self, and separation/distance were designated as MS and linked with maladaptive coping (Katsampouris et al., 2020).

Stress research has been critiqued regarding some of the traditional methods (e.g., self‐report questionnaires) that are employed to measure and examine psychological stress (Slavich, 2019). Explicit self‐report measures of stress have been seen as vulnerable to bias effects, such as social desirability concerns, faking, and retrospective call (Egloff & Schmukle, 2003; Fiedler, Messner, & Bluemke, 2006; Yoshiuchi, Yamamoto, & Akabayashi, 2008). Individuals are likely to consciously alter some of their questionnaire responses and underrate stress reported due to social desirability (Sato & Kawahara, 2012). Despite some recent innovative methods that have been used to assess stress (Zhong et al., 2020), relatively little attention has been drawn to understand stress and health outcomes at a cognitive, unconscious, and innate level (Cheetham, Turner‐Cobb, & Gamble, 2016). To date, only one study has specifically focused on AS and MS and the ability of adults to cope, and this examined explicit understanding (Katsampouris et al., 2020). Implicit understanding of the association between AS and MS and the ability of adults to cope has so far received no attention.

In psychological research, the implicit association test (IAT) has been used extensively including for the assessment of health‐related concepts; for example, to measure preference for a group (e.g., psychology or chemistry) (Nosek, 2005); beliefs, stereotypes, or prejudice (e.g., males/females and maths/arts) (Nosek, Banaji, & Greenwald, 2002); gender/ethnic identity and implicit bias (Devos & Banaji, 2005; Egloff & Schmukle, 2002; Greenwald & Farnham, 2000); self‐esteem (e.g., self/other and good/bad) (Greenwald & Farnham, 2000); individual attitudes towards health‐related behaviours such as smoking, alcohol, and diet (Ames et al., 2013; Andrews, Hampson, Greenwald, Gordon, & Widdop, 2010; Palfai & Ostafin, 2003; Perugini, 2005); and children’s implicit understanding of the stress and illness relationship (Cheetham et al., 2016). Additionally, biological markers of stress (i.e., hair cortisol concentration) have been strongly associated with implicit stress and weakly associated with explicit perceived stress (Geng, Xiang, Yang, Shen, & Sang, 2016).

Yet, there has been limited research around the IAT regarding stress and the assessment of stress via an implicit measure has provided some contradictory findings possibly as a result of the nature of stress manipulation in the studies. Although the IAT has been a valid measure of trait anxiety (Egloff & Schmukle, 2002), state anxiety was assessed using an IAT employing stimuli from self/other categories and anxiety/calmness categories (Schmukle & Egloff, 2004). The IAT did not reveal an implicit effect from an experimentally induced state anxiety due to a stressful public speaking task neither on the experimental nor on the control group. No association has been found between implicitly and explicitly examined anxiety with recognition of and brain response to facial emotions (Suslow et al., 2019). The IAT has not previously been applied to assess unconscious conceptualization of stress and ability to cope in adults. This is the first study to experimentally explore an implicit distinction between AS and MS and their unconscious association with coping ability.

The aim of this study was to examine whether implicit associations exist between AS and MS and coping appraisal in a sample of young through middle‐aged adults. It was hypothesized that (1) explicit associations would be revealed between AS and ability to cope, and between MS and inability to cope; (2) adults would implicitly associate with a faster reaction time (RT) and higher accuracy consistent pairs (AS/ability to cope; MS/inability to cope) rather than inconsistent pairs (AS/inability to cope; MS/ability to cope) (using the conventional IAT scoring algorithm); and (3) adults would implicitly indicate a stronger preference for consistent than inconsistent pairs.

## Methods

Data were collected from adults between September and October 2017, using a quantitative design composed of two parts. Firstly, a computer‐based implicit measure (IAT) to compare RT and accuracy for consistent and inconsistent pair responses (AS/MS and ability/inability to cope); and secondly a self‐report questionnaire to assess adults’ explicit understanding of the AS/MS distinction. Full ethical approval was granted for this study by the University Departmental Ethics Committee (#17‐202).

### Participants

One hundred and fifteen participants were recruited using snowball sampling via social media and word of mouth, across the south west area of England. Of those, 100 adult participants (*M* = 28.27 years, *SD* = 10.02, range: 18–58 years) (75 females) consented to participate in this study, which took place in the Department of Psychology laboratories, and passed the eligibility criteria. The majority of the sample were white (76%), single (36%) or in a relationship (35%), students (49%) or employed (47%), and having obtained a degree (36%). Participants excluded were those aged under 18 years; those with any stress‐related condition as far as they were aware; and those having no uncorrected visual abnormalities (e.g., colour blindness).

### Measures

Questions assessed demographic variables: age, sex, ethnicity, marital status, educational attainment, employment status.

In order to assess innate associations between stress and coping appraisal using more objective measures, the IAT was employed to examine cognitive unconscious attitudes and/or beliefs that individuals may not be willing or able to explicitly report (Cvencek, Greenwald, & Meltzoff, 2011). The IAT has been considered as an established computer‐based cognitive assessment tool, which examines the strength of automatic unconscious, unintentional, uncontrollable mental associations between concepts and evaluations or stereotypes and attitudes towards age, gender, and race measuring RT, rather than conscious, deliberate, and controlled associations (Fazio & Olson, 2003; Greenwald, McGhee, & Schwartz, 1998). In this study, adults’ implicit perceptions of the link between AS and MS and the ability to cope was examined using this experimental paradigm. Employing the IAT, the assumption has been that consistent pairs (e.g., AS and ability to cope) would be performed more accurately and faster than pairing inconsistent combinations (e.g., MS and ability to cope).

Implicit associations between AS/MS and coping were measured using a modified IAT adapted from the original IAT version (Greenwald et al., 1998) and measures RT and accuracy to stimulus items. The IAT has shown better predictive validity than explicit measures, with a reported Cronbach’s α of .7 to .9 that is higher than other latency‐based measures (Greenwald & Nosek, 2001; Greenwald, Poehlman, Uhlmann, & Banaji, 2009; Nosek, Greenwald, & Banaji, 2007). Participants were asked to sort the stimulus items into one of four categories using two response buttons, where each response button corresponds to two categories. This enables the assessment of associations between a target and an attribute concept by measuring whether people are faster and more accurate to respond when consistent/associated or inconsistent/unassociated pairs are paired on the same response button. Faster RT indicates a stronger link between concept and attribute. Support for obtaining explicit measures alongside implicit measures is justified through the ability to obtain a balanced design for the concepts of interest and to compare the findings between the explicit and implicit assessments (Greenwald et al., 2002; Nosek, Greenwald, & Banaji, 2005).

Explicit associations between AS/MS and coping were evaluated using two measurements. Firstly, the Life Events Inventory (LEI) assessed the occurrence of and emotional distress level experienced from a wide range of desirable or undesirable life events relating to the last year (Tennant & Andrews, 1976). Participants were asked to read each statement of a modified 15‐item LEI and to indicate for each event that had occurred, how stressful their experience of the event had been. The modified LEI consisted of nine life event stressors that fit the criteria for AS and six life event stressors that fit the criteria for MS. Four members of the research team independently evaluated and selected the most appropriate for this study ancient/modern life event questionnaire items from those that had been used in previous studies by Katsampouris et al. (2020) and Schreier and Evans (2003), until agreement was reached. Participants scored each event on a 8‐point Likert scale from 0 (*not happened*), 1 (*happened but not at all stressful*) to 7 (*happened and extremely stressful*). The total number and mean severity score of life events were computed for each participant; Cronbach’s α = .71 for this sample. Secondly, participants were asked to describe how they had coped with each one of these life events or how they considered they would have coped, in cases where they had not experienced the stressor in the last year. Participants used an open text box to describe how they would or did cope with life event stressors.

These coping descriptions were transformed through discussion and consensus by the research team into 15 new quantifiable life event stressor variables; nine for AS (i.e., ‘new_AS_cat_1’, ‘new_AS_cat_2’, …, ‘new_AS_cat_9’) and six for MS (i.e., ‘new_MS_cat_1’, ‘new_MS_cat_2’, …, ‘new_MS_cat_6’). The 15 quantitative categorical variables were then coded as 0 (maladaptive coping inability) and 1 (adaptive coping ability), based on the recommendations for categorization of quotes as adaptive/maladaptive coping given in Katsampouris et al. (2020).

This yielded two new categorical variables for each participant (i.e., ‘new_AS_cat_score’ and ‘new_MS_cat_score’) from which an average coping ability score was computed for each participant. An average score of 0 indicated maladaptive coping ability (scores < 0.5 were rounded to 0), and an average score of 1 indicated adaptive coping ability (scores > 0.5 were rounded to 1). Based on the ‘new_AS_cat_score’ and ‘new_MS_cat_score’ variables, a final categorical variable was computed and coded as 0 (*no ancient and modern stressor distinction*) and 1 (*ancient and modern stressor distinction*) to enable the explicit association score between ancient/modern stressor and coping and to test hypothesis (iii). Participants with either 0’s or 1’s in both the ‘new_AS_cat_score’ and ‘new_MS_cat_score’ variables were assigned with the code value 0 in the final variable, implying no ability to distinguish between AS and MS.

### Procedure

Participants were given detailed information about the study through a participant information sheet prior to obtaining written consent. Completion of both the implicit and explicit measures lasted for about 45 minutes. A debrief sheet was provided at completion of the study to explain the concept of AS and MS. What differentiates the IAT from other implicit measures (e.g., priming tasks) is that it requires an unambiguous categorization of target items (e.g., words, pictures) to concept categories (Nosek et al., 2005). The IAT typically involves 180 trials (Greenwald, Nosek, & Banaji, 2003). Being the first study to explore AS and MS using an IAT, this study employed 208 trials in total, 96 of which were critical trials, with an intertrial interval of 400 ms. Participants were provided with image stimuli selected by the researchers to represent life events but not with the intention to induce stress. In an IAT, a trial is the presentation of a single stimulus (image, word) that requires categorization. The stimuli in this study were a combination of images and words that were presented in the centre of a computer screen and were related to the four target concept categories of: ‘old problems’ (AS), ‘new problems’ (MS), ‘having sufficient resources’ (adaptive coping ability), and ‘not having sufficient resources’ (maladaptive coping inability).

Thirty‐eight stimulus images (all coloured, non‐cartoon) for the stressors were selected from a combination of copyright‐free image websites. Thirty‐eight stimulus words for coping were selected from the Oxford English Dictionary and from previous research (Katsampouris et al., 2020). Seven Health Psychology researchers rated the strength of association between these stimuli and the four target concepts. The highest rated 24 images (representing the ancient‐designated life event stressors of death/bereavement, movement, health/illness of others and social/interpersonal arguments, and the modern‐designated life event stressors of unemployment, financial problems, health/illness of self, and separation/distance) and 24 words were used as stimulus items (12 words and 12 images per category) (see Table 1). A pilot study was conducted with 10% of the total sample to judge the appropriateness of stimuli analysing RT (*p* < .001) and accuracy (*p* = .004) scores for consistent and inconsistent pairs via paired samples *t*‐tests. The overall consensus criterion on the stimuli’s association with the four target concepts showed a substantial inter‐rater reliability agreement of κ = .71, *p *< .001, 95% CI [0.664, 0.762] (Fleiss, Levin, & Paik, 2003).

**Table 1:** Included words in IAT coping ability categories

Adaptive coping ability (‘Having sufficient resources’)	Maladaptive coping inability (‘Not having sufficient resources’)
Deal with	Struggle
Manageable	Unmanageable
Adaptable	Unresolved
Resolved	Uncontrollable
Overcome	Mismanage
Handle	Unfeasible
Controllable	Inflexible
Doable	Mishandle
Effective	Ineffective
Functional	Dysfunctional
Flexible	Maladaptive
Feasible	Unfavourable

IAT performance was measured using response latency (the speed of the response or RT) and response accuracy (whether the responses were correct or incorrect) to each stimulus item. Accuracy of response to each stimulus was recorded by ePrime Professional 2.0 and coded as 0 and 1 for incorrect/inaccurate response and correct/accurate response, respectively. Accuracy of responses refers to how many trials participants responded to correctly; the closer the mean score was to 1, the more correct/accurate responses were. A mean score for accuracy of responses for consistent and inconsistent pairs was computed for each participant.

A computer running ePrime was used to display the stimuli, and participants responded using a response button (only two response keys were needed). Stimulus items were presented in a random order generated by ePrime and each item was presented once. The 48 stimulus items in the IAT were presented in seven blocks: Blocks 1 and 5 contained 24 AS and MS images, block 2 contained 24 adaptive coping ability and maladaptive coping inability words, and critical blocks 4 and 7 included all 48 items. Only two categories were shown in blocks 1, 2, and 5; therefore, each response button corresponded to one category. In blocks 4 and 7, four categories were shown, two categories per response button to assess implicit associations of AS/MS and coping, when the two concepts AS/adaptive coping ability and MS/maladaptive coping inability are paired together (i.e., consistent pairs) or conversely paired (i.e., inconsistent pairs: AS/maladaptive coping inability and MS/adaptive coping ability). In the consistent pair block 4, the categories AS/adaptive coping ability were shown on the same side of the screen and shared a response button, and the categories of MS/maladaptive coping inability were shown on the other side of the screen and shared a response button. In the inconsistent pair block 7, AS/maladaptive coping inability were paired together and MS/adaptive coping ability. Prior to the critical blocks 4 and 7, practice blocks 3 and 6 included 20 practice trials each (Greenwald et al., 2003).

All instructions were given on screen, and during all seven blocks, a notice of ‘Press the e key for’ and ‘Press the i key for’ with the two or four categories remained on the screen as a reminder to participants as to which button corresponds to which category. Two response buttons corresponded to a category: the left ‘e’ button to the category on the left side of the screen and the right ‘i’ button to the category on the right side. The participants used these response buttons to indicate which category the stimulus word or image belonged to. If a participant had given an incorrect response, they saw an error message and moved straight on the next trial.

Counterbalancing ensured that half participants were randomly allocated the consistent pairs first (order A) and half were presented with the inconsistent pairs first (order B) (Cvencek et al., 2011). The AS and MS categories alternated between the left and right positions on the screen (blocks 1 and 5) in order to minimize the effects of practice (Nosek et al., 2005), whereas the ability to cope and inability to cope categories remained unchanged throughout the test to cause minimal confusion to participants (see Table 2). The IAT score depends upon how long it takes a person typically to categorize the stimuli in the fourth block versus the seventh block.

**Table 2:** Sequence of trial blocks in the ancient and modern stressor IAT

Block	Number of trials	Function	Items assigned to the left‐key response	Items assigned to the right‐key response
1	24	Practice	Old problems images	New problems images
2	24	Practice	Having sufficient resources words	Not having sufficient resources words
3	20	Practice	Old problems images + Having sufficient resources words	New problems images + Not having sufficient resources words
4	48	Test	Old problems images + Having sufficient resources words	New problems images + Not having sufficient resources words
5	24	Practice	New problems images	Old problems images
6	20	Practice	New problems images + Having sufficient resources words	Old problems images + Not having sufficient resources words
7	48	Test	New problems images + Having sufficient resources words	Old problems images + Not having sufficient resources words

Note

For half the subjects, the positions of blocks 1, 3, and 4 are switched with those of blocks 5, 6, and 7. The procedure in blocks 3, 4, 6, and 7 is to alternate trials that present either an adaptive or maladaptive coping word with trials that presented either an ancient or modern stressor image. These strategies were used to reduce the typical effect of order in which the two combined tasks are performed.

### Analytical plan

Explicit associations were assessed through chi‐square test and paired samples *t*‐tests in IBM SPSS Statistics software v.22. Implicit associations were assessed through repeated‐measures ANCOVAs (sex and age were used as covariates) to compare RT and accuracy scores for consistent and inconsistent pair responses. The data were also analysed through univariate ANCOVA (sex, age, and block order were used as covariates) using the improved *D* scoring algorithm rather than RT scores (Greenwald et al., 2003). This additional *D* score analysis is presented for consistency and comparison with the conventional IAT measure. *D* scores represent the difference between the mean RT scores for consistent and inconsistent pairs and the variance of the within‐blocks response latencies; a positive *D* score suggests an implicit preference for the consistent pairs (equivalent to faster RT and greater accuracy to those pairs) and a negative score indicates a preference for the inconsistent pairs (Greenwald et al., 1998, 2003).

## Results

### Descriptive analyses

Mean scores are indicated for RT and accuracy scores for consistent and inconsistent pairs, and AS and MS on the IAT (see Table 3). Adults responded faster and more accurately to consistent than inconsistent pairs.

**Table 3:** Means, standard deviations (*SD*), and range (minimum–maximum) of IAT scores and psychological variables in adults (*N* = 100)

Measures	Mean (*SD*)	Range: min‐max
IAT scores		
RT (ms) Consistent pairs	948.67 (281.52)	452.25 to 1,622.29
RT (ms) Inconsistent pairs	1,629.98 (457.76)	729.93 to 2,702.65
Accuracy Consistent pairs	0.79 (0.11)	0.50 to 0.98
Accuracy Inconsistency pairs	0.75 (0.13)	0.34 to 0.96
*D* score	0.14 (0.60)	−.98 to 1.93
Psychological variable scores		
Total number Ancient stressors & Adaptive coping ability	5.14 (3.73)	0 to 9.00
Mean severity Ancient stressors & Adaptive coping ability	1.48 (1.79)	0 to 7.00
Total number Modern stressors & Maladaptive coping inability	2.96 (2.57)	0 to 6.00
Mean severity Modern stressors & Maladaptive coping inability	2.40 (2.00)	0 to 9.00

### Main effects

#### Explicit analyses

A chi‐square test for association was conducted between AS/MS and coping ability; there was a statistically significant weak association, χ^2^(1) = 9.78, φ = .22, *p* = .002, whereby AS were associated with better coping ability and MS with the inability to cope.

To assess an explicit AS/MS distinction, a paired samples *t*‐test was conducted comparing the total number of AS and MS. There was a significant difference in the scores for AS (*M* = 5.14, *SD* = 3.73) and MS (*M* = 2.96, *SD* = 2.57); *t*(99) = 12.43, *p* < .001, 95% CI [1.83, 2.53] with a medium effect size (Cohen’s *d* = .07) indicating that adults coped with more AS than MS. A paired samples *t*‐test was conducted comparing the mean severity of AS and MS. There was a significant difference in the severity scores for AS (*M* = 1.48, *SD* = 1.79) and MS (*M* = 2.40, *SD* = 2.00); *t*(99) = −6.49, *p* < .001, 95% CI [−1.20, −0.639] with a medium effect size (Cohen’s *d* = −.48) indicating that adults experienced MS more stressful than AS.

Pearson’s *r* correlations revealed no significant associations between the explicit and implicit measures implying that both measures are distinct and divergent from each other (see Table 4).

**Table 4:** Correlations between explicit and implicit variables (*N* = 100)

Explicit variables	Implicit variables				
	RT		Accuracy		*D* scores
	Consistent pairs	Inconsistent pairs	Consistent pairs	Inconsistent pairs	
Total number Ancient stressors & Adaptive coping ability	.014	−.012	.028	.032	.039
Mean severity Ancient stressors & Adaptive coping ability	−.045	.011	−.116	.150	−.012
Total number Modern stressors & Maladaptive coping inability	.033	−.043	.079	−.029	−.013
Mean severity Modern stressors & Maladaptive coping inability	−.068	−.012	−.100	.126	−.096

Note

Figures show Pearson’s *r* correlation coefficients between explicit and implicit variables.

#### Implicit analyses

To assess an implicit AS/MS distinction, a repeated‐measures ANCOVA indicated a main effect of RT *F*(1, 97) = 4.06, *p* = .047, partial η^2^ = .04 such that RT for consistent pairs (*M* = 948.67 ms, *SD* = 281.52) was faster than for inconsistent pairs (*M* = 1629.98 ms, *SD* = 457.76).

A repeated‐measures ANCOVA indicated a main effect of accuracy *F*(1, 97) = 4.40, *p* = .039, partial η^2^ = .04 such that accuracy for consistent pairs (*M* = 0.79, *SD* = 0.11) was higher than for inconsistent pairs (*M* = 0.75, *SD* = 0.13). Sex and age were not significant as main effects controlling for them in both repeated‐measures ANCOVAs.

*D* scores were analysed using a univariate ANCOVA indicating neither a main effect of explicit measure (*p* = .797) nor indirect effects of age (*p* = .063) and order of blocks (*p* = .053). Sex revealed an indirect effect; *F*(1, 95) = 9.17, *p* = .003, partial η^2^ = .09. Independent *t*‐test revealed that males (*M* = 0.43, *SE* = .07) showed a stronger preference for consistent pairs than females (*M* = .05, *SE* = .07), *t*(71.84) = 3.65, *p* < .001, 95% CI [0.173, 0.590] with a medium to large effect size (Cohen’s *d* = .73) indicating that males than females associated faster and more accurately AS and coping ability than MS and coping inability. Males showed a stronger implicit AS/MS distinction than females.

## Discussion

This study set out to assess the feasibility of distinguishing between ancient and modern stressors at an implicit, cognitive, and unconscious level in adult population sample. This study built upon emerging evidence of an explicit distinction between AS and MS to test implicit associations using an experimental paradigm. Firstly, it was hypothesized that AS would explicitly be associated with the ability to cope and MS with the inability to cope. Support was found for explicit associations between AS and ability to cope and MS and inability to cope, which is congruent with previous findings of an explicit AS/MS distinction (Katsampouris et al., 2020). The explicit associations found in this study are in line with the theory that established adaptive psychophysiological coping mechanisms enable people to cope with AS in contrast with more recently evolved MS (Schreier & Evans, 2003).

Quantitative analyses of the explicit measures found that AS were associated with coping ability and MS with coping inability. These analyses provided evidence that this group of young to middle‐aged adults coped with a greater total number of AS than MS and that they experienced MS as more stressful than AS, which is consistent with the findings of mixed‐methods studies in younger and older adults (Katsampouris et al., 2020). The concepts of interest in this study were not subject to social acceptability; thus, findings were less likely to have been impacted by issues of self‐representation, social desirability, retrospection, or participant faking and more likely to reveal an explicit association (Fiedler et al., 2006; Gawronski, Hofmann, & Wilbur, 2006; Hofmann, Gawronski, Gschwendner, Le, & Schmitt, 2005; Yoshiuchi et al., 2008). There were no associations found between the explicit and implicit measures, which implies that both measures have distinct divergent characteristics, are completely empirically independent from each other and involve different processes (Egloff & Schmukle, 2003; Greenwald et al., 1998).

Secondly, it was hypothesized that participants would reveal faster RTs and greater accuracy for consistent pairs (AS/ability to cope; MS/inability to cope) rather than inconsistent pairs (AS/inability to cope; MS/ability to cope) using the conventional IAT scoring algorithm for analysis purposes; and that they would show a stronger preference for consistent than inconsistent pairs using the improved *D* scoring algorithm. Overall, support was found for implicit associations between AS/ability to cope and MS/inability to cope, which implies an implicit distinction since participants associated faster and more accurately the consistent pairs than inconsistent pairs. Analyses of the implicit AS/MS distinction found main effects of RTs and accuracy, indicating that RT and accuracy for consistent pairs were faster and higher, respectively, rather than for inconsistent pairs. Supportive evidence for the implicit AS/MS distinction was provided by the alternative *D* score analysis. In this alternative analysis, findings with our adult sample revealed an unconscious stronger preference for consistent than inconsistent pairs.

Regarding the RT and accuracy score analyses, results imply that it might have been easier for participants to link stimuli when associated pairs shared a response button. This could be because people might be more capable in an evolutionary sense to adapt and cope with AS, rather than with MS that require more time and physiological energy to cope, possibly resulting in a greater stress response, allostatic load, and impact on physical health (McEwen, 1998, 2007; Schreier & Evans, 2003). An alternative explanation might suggest that less conscious cognitive processes enable people to innately cope with familiar AS that have been around for many years (Leary, Adams, & Tate, 2006).

Regarding the alternative analysis, these implicit associations have been further supported using the *D* scores, with no main effect found for explicit measure implying that the implicit and explicit measures have been distinct and no indirect effect found for order of blocks indicating that counterbalancing did not affect the *D* score data. Additionally, there was no indirect effect of age on the implicit associations, which might have been expected because of the specific age group that participated. As the RT and *D* scores are drawn from the same data, it was expected these *D* score data to show similar patterns in findings with the RT score data. However, an indirect effect of sex was found on *D* score analyses indicating that adult males than females implicitly associated faster and more accurately AS, rather than MS, with ability to cope showing a stronger implicit preference for consistent pairs than inconsistent pairs.

As hypothesized, these adult participants made implicit associations indicating an unconscious AS/MS distinction. Specifically, males, rather than females, showed a stronger implicit AS/MS distinction. Although previous research on the explicit distinction did not reveal any gender differences in coping with AS and MS (Katsampouris et al., 2020), the current findings suggest that sex differences might exist at an unconscious implicit level. One explanation may be that participants have not been consciously, introspectively aware of the concepts of interest and might have been more willing and able to report their true attitudes and beliefs compared to those at a more conscious explicit level (e.g., in self‐report questionnaires) (Cvencek et al., 2011; Greenwald & Banaji, 1995; Greenwald et al., 1998, 2003).

These findings extend recent work on an explicit chronological distinction between AS and MS to an unconscious distinction. They suggest an important taxonomy for understanding how individuals cope with stress with implications for improving health outcomes. More broadly, the present findings provide additional evidence that the psychosocial characteristic of adaptive/maladaptive coping could be regarded as one of the five stressor characteristics to view the AS/MS distinction along a continuum (Katsampouris et al., 2020).

Despite the application of an experimental paradigm to an innovative methodological assessment of the AS/MS distinction, the present findings should be regarded as suggestive rather than conclusive, and we acknowledge a number of limitations. Adult participants were mainly recruited from the south‐west region of England, with limited geographical heterogeneity and future work would benefit from a more diverse recruitment. Although this study attempted to invite participants across the wider adulthood spectrum for taking part, a specific age group was recruited, that of young to middle‐aged adults. Future research is called for to closely assess differences across age groups, and perhaps more specifically between young and older adults. Previous work on AS and MS (Katsampouris et al., 2020) did not reveal any gender or age differences on AS and MS at an explicit level, yet this study found some sex differences between AS and MS at an implicit level. It is likely that participants reported true attitudes and beliefs through an implicit assessment rather than via explicit self‐report questionnaires. Such differences could provide evidence for adult groups regarding the impact of perceived ancient/modern stress on health.

Regarding methodological challenges, word and image stimuli were carefully selected to be explicitly distinguishable from each other and to best represent only one of the categories of ‘old problems’, ‘new problems’, ‘having sufficient resources’ and ‘not having sufficient resources’ as counterparts to ‘AS’, ‘MS’, ‘ability to cope’, and ‘inability to cope’, respectively (Nosek et al., 2005). Issues relating to the application of this IAT paradigm to the AS and MS might have included a lack of uniformity, mental representation, and conceptual meaning of images in individuals from various socio‐cultural backgrounds. Despite this, these stimulus descriptions served their purpose as comprehensible and opposing categories to the concepts of interest.

Consideration of other method‐related issues (e.g., order of measures and blocks, IAT trials, practice trials, number of stimuli per category, previous IAT experience, fixed or random stimuli presentation, counterbalance) was provided by additionally using the improved *D* scoring algorithm (Fiedler et al., 2006; Greenwald & Nosek, 2001; Nosek et al., 2005). The improved algorithm has been seen as a better measurement of implicit associations than the conventional IAT score, as it computes the difference and variance between and within the block latencies (i.e., error measurement). However, we are aware of some debate about its scoring comparisons because it statistically standardizes both the mean and the variance of scores (Greenwald et al., 2003). Extraneous factors that could be considered in future research include participant cognitive fluency, self‐representation concerns, faking, and introspection (Egloff & Schmukle, 2003; Fiedler et al., 2006; Greenwald, 2004). Participants in this study however, were not aware of the AS and MS concept. This IAT paradigm did not appear to cause any discomfort or unwillingness to participants which might have prevented them from reporting true beliefs (Sato & Kawahara, 2012).

Although the IAT has been employed in several psychological research fields, there has been comparatively little work applying it as a technique within stress research (Cheetham et al., 2016; Geng et al., 2016). To ensure a reliable level of interpretation of the present findings, this IAT was designed based on published paradigm guidelines and was analysed via the conventional and improved scoring, both of which provided consistent findings.

The use of the well‐established IAT paradigm to assess the feasibility of distinguishing between AS and MS has proved valuable as it moves away from the traditional explicit self‐report measures, although its application to the AS/MS and coping concepts needs further examination. This study highlights the importance of exploring implicit associations in an adult population in order to holistically understand implicit associations between psychosocial stressors and coping ability across adulthood. The findings of this study provide some evidence to develop a new chronological taxonomy of stress, with further examination of age‐specific characteristics relating to this taxonomy, the impact of which requires further assessment with application to health outcomes.

## Conclusion

This research was the first to employ an experimental implicit paradigm to explore whether adults could unconsciously distinguish between ancient and modern stressors. It found support for previous research suggesting an explicit AS and MS distinction regarding established adaptive psychological coping mechanisms; that of adults are more able to adapt and cope with familiar AS rather than with more MS. This study provided some significant empirical evidence, using both the traditional and alternative improved paradigm scorings, that adults implicitly associated faster and more accurately consistent pairs (AS/ability to cope; MS/inability to cope) rather than inconsistent pairs (AS/inability to cope; MS/ability to cope). The current findings have assessed both implicit and explicit methods suggesting a novel chronological taxonomy of psychosocial life event stressors with associated coping profile. Results highlight the benefits of using experimental research paradigms to complement more traditional methods in stress research. Future research is called for to systematically explore this notion of an implicit AS and MS distinction and how it advances our current understanding of stress, with potential for inclusion in development of psychosocial interventions.

## Data availability statement

The data that support the findings of this study are available from the corresponding author upon reasonable request.

## Author contributions

**Rachel S. Arnold** (Methodology; Supervision; Validation; Writing – review & editing) **Julie C. Barnett** (Methodology; Supervision; Validation; Writing – review & editing) **Evangelos Katsampouris** (Conceptualization; Data curation; Formal analysis; Investigation; Methodology; Project administration; Resources; Validation; Visualization; Writing – original draft; Writing – review & editing) **Julie M. Turner‐Cobb** (Conceptualization; Methodology; Project administration; Supervision; Validation; Visualization; Writing – original draft; Writing – review & editing).

## Conflicts of interest

The author(s) declared no potential conflicts of interest with respect to the research, authorship and/or publication of this article.

## Funding

The author(s) received no financial support for the research, authorship and/or publication of this article.
